# Design and implementation of a clinical laboratory information system in a low-resource setting

**DOI:** 10.4102/ajlm.v8i1.841

**Published:** 2019-10-28

**Authors:** Timothy M. Mtonga, Faheema E. Choonara, Jeremy U. Espino, Chimwemwe Kachaje, Kenneth Kapundi, Takondwa E. Mengezi, Soyapi L. Mumba, Gerald P. Douglas

**Affiliations:** 1 Department of Biomedical Informatics, School of Medicine, University of Pittsburgh, Pittsburgh, Pennsylvania, United States; 2 Kamuzu Central Hospital, Lilongwe, Malawi; 3 Baobab Health Trust, Lilongwe, Malawi

**Keywords:** low-resource setting, laboratory testing, laboratory information system, Malawi, informatics interventions

## Abstract

**Background:**

Reducing laboratory errors presents a significant opportunity for both cost reduction and healthcare quality improvement. This is particularly true in low-resource settings where laboratory errors are further exacerbated by poor infrastructure and shortages in a trained workforce. Informatics interventions can be used to address some of the sources of laboratory errors.

**Objectives:**

This article describes the development process for a clinical laboratory information system (LIS) that leverages informatics interventions to address problems in the laboratory testing process at a hospital in a low-resource setting.

**Methods:**

We designed interventions using informatics methods for previously identified problems in the laboratory testing process at a clinical laboratory in a low-resource setting. First, we reviewed a pre-existing LIS functionality assessment toolkit and consulted with laboratory personnel. This provided requirements that were developed into a LIS with interventions designed to address the problems that had been identified. We piloted the LIS at the Kamuzu Central Hospital in Lilongwe, Malawi.

**Results:**

We implemented a series of informatics interventions in the form of a LIS to address sources of laboratory errors and support the entire laboratory testing process. Custom hardware was built to support the ordering of laboratory tests and review of laboratory test results.

**Conclusion:**

Our experience highlights the potential of using informatics interventions to address systemic problems in the laboratory testing process in low-resource settings. Implementing these interventions may require innovation of new hardware to address various contextual issues. We strongly encourage thorough testing of such innovations to reduce the risk of failure when implemented.

## Introduction

Laboratory testing plays a vital role in clinical decision-making. It is estimated that up to 70% of medical decisions in high-resource healthcare settings are made based on clinical laboratory test results.^1,2^ Even though access to clinical laboratory services is comparatively lower in low-resource settings, studies show that clinicians in low-resource settings also make most decisions based on laboratory testing.^3,4^ Despite the importance of laboratory test results in clinical decision-making, little effort has been made in low-resource settings to improve the entire laboratory testing process, which starts when the test is first ordered and ends when the results are interpreted and a clinical decision is made.^5^

Laboratory errors include a wide variety of mistakes in the testing process and have no universally accepted definition. We define a laboratory error as any event or mistake that leads to failure to perform a laboratory test, misdiagnosis of a laboratory test, or delayed reporting of laboratory test results. In 2001, it was estimated that laboratory errors accounted for $200 million – $400 million in American healthcare expenditures per annum.^6^ Since then, the rate of utilisation of laboratory services has increased, making the reduction of laboratory errors a significant opportunity for cost reduction and healthcare quality improvement.

Recent studies have tried to categorise errors using phases of the total testing process, which comprises pre-analytical, analytical and post-analytical phases.^7^ The pre-analytical phase covers all activities from when the test is ordered to when the specimen is delivered to the laboratory for testing. The analytical phase covers the activities involved in the actual testing of the specimen and the post-analytical phase involves the reporting and interpretation of the laboratory result. Among the phases of the total testing process, it has been observed that most laboratory errors happen outside of the analytical phase.^8^ An example of an error outside the analytical phase is the mislabelling of a specimen, which could happen during the drawing of a sample in the pre-analytical phase. While error rates vary between health facilities, it is estimated that 32% – 75% of all laboratory errors happen in the pre-analytical phase.^9^ Error rates in the analytical phase are estimated in the range of 13% – 32% and in the post-analytical phase in the range of 9% – 31%.^9^

Informatics interventions may be useful in reducing such laboratory errors. Examples of such interventions are computer-aided ordering of laboratory tests, barcode labelling of specimen tubes, and automating the reporting of laboratory test results. These interventions are often provided using computer systems that allow physicians to order diagnostic tests, medications, and other procedures, commonly referred to as computerised provider order entry.^10^ Computerised provider order entry is often a part of a larger electronic health record system. However, such comprehensive electronic health record systems have low penetration in low-resource settings where the burden of disease is high and laboratory errors are further exacerbated by poor infrastructure, shortages in trained workforce and informational challenges.^1,11^

Although laboratory information systems (LIS) have been shown to help reduce laboratory errors, little information is available on the implementation of these in low-resource settings. Furthermore, most descriptions of LIS implementations in low-resource settings focus on the analytical phase of the total testing process.^12^ In this article, we describe preliminary work in developing a LIS that addresses problems using informatics interventions to support all phases of the total testing process in a low-resource setting with no pre-existing computerised provider Order Entry system.

## Methods

### Ethical considerations

This article followed all ethical standards for research without direct contact with human or animal subjects.

### Setting

We implemented a LIS at the Kamuzu Central Hospital (KCH) in Lilongwe, Malawi, between January and March 2015. The Kamuzu Central Hospital is a 750-bed government-operated referral hospital. The laboratory at KCH comprises eight departments: microbiology, parasitology, serology, haematology, molecular biology, blood bank, flow cytometry and biochemistry. These departments perform laboratory tests for both outpatients and inpatients at the hospital and conducted 242 242 tests between 01 July 2010 and 30 June 2011.^13^ The system described in this article was piloted in the outpatient tuberculosis clinic of the hospital and the microbiology department of the laboratory starting 31 March 2015.

### User requirements and system capabilities

Requirements for the LIS were provided by laboratory technicians in the form of user stories. A user story is a succinct way of representing a task that a user will want to perform using an information resource.^14^ It includes the role of the user, the task or action and the benefit, goal or achievement. An example of a user story in this context is:

As a laboratory technician, I want to know which specimen was drawn first so that I can prioritise it for analysis to reduce the number of non-viable specimens.

We compiled a consolidated list of user stories for each phase of the total testing process. We used that list to define a set of functionality requirements from the laboratory technicians.

To ensure that no core functionality was omitted from the specifications, we leveraged the Laboratory Information System Functionality Assessment Toolkit (LIS-FAT) developed by the Association of Pathology Informatics. This assessment toolkit provides 850 declarative statements that describe the functions that a LIS should possess.^15^ An example of a functionality statement from LIS-FAT is:

A laboratory information system should provide intelligent sample labelling that groups samples based on the test to be done and prints them out.

The LIS-FAT was originally intended for use as a LIS evaluation checklist. However, in our implementation, we repurposed it to define capabilities for the proposed system. Furthermore, we recognised that the LIS-FAT was primarily developed for use in a setting with adequate resources and some aspects of it may not be well suited for a low-resource setting. We therefore assessed the LIS-FAT functionality statements and selected those that focused on direct user needs and were most applicable in a low-resource setting. Special effort was made to ensure that major functional categories of the LIS-FAT were not overlooked. This resulted in a customised LIS-FAT applicable to a low-resource setting, with the declarative statements describing the core requirements for LIS in this setting.

To elucidate the dependencies that could drive the design phase, all functionality statements created in this step were grouped into high, medium, and low priority categories by a group of laboratory management personnel. This helped determine the order in which the functionality would be implemented to ensure that the most important functionality was implemented first.

### System design and development

Laboratory information system software can be commercial, open-source, or home-grown. We chose to build on existing open-source LIS software and customise it based on our functional requirements. Before any functionality was implemented, we conducted a design validation study of two open-source LIS software systems to determine the extent to which they implemented the required functionality for the KCH laboratory.^16^ These systems were Open Enterprise Laboratory Information System (OpenELIS) and Basic Laboratory Information System (BLIS).^12,17^ We assessed and ranked the systems based on the number of functionality requirements that they satisfied for the LIS implementation at KCH. A functionality requirement was considered satisfied if the LIS had a feature that could be used to achieve the goal of that requirement. The choice between the systems was based on the total number of required functions that each of the systems possessed. The system with the most functionality requirements was selected as the foundation upon which the LIS implementation at KCH would be built, and a comprehensive design was made around it to ensure that all functional requirements were met.

We also realised that information systems frequently emphasise the collection and use of data for reporting purposes and streamlining workflow. However, the use of such systems does not necessarily result in improved outcomes. To maximise the value of the LIS, we considered problems identified in the laboratory testing process and described in previous publications.^11,13^ Targeted informatics interventions were developed and incorporated into the system’s design to address each of these problems.

Upon completion of the system design, a team of three developers (C.K., K.K., T.M.M.) iteratively implemented and integrated the remaining functionality over eight weeks from mid-January to mid-March 2015. During this time, clinicians and laboratory personnel provided initial feedback which we used to refine the user interfaces for the new features.

## Results

### User requirements and system capabilities

A list of 34 user stories was compiled and mapped into functionality statements for the KCH LIS implementation. An additional 41 statements were added from our review of the LIS-FAT statements. The selected LIS-FAT statements had direct user benefits in keeping with the user stories provided by laboratory technicians and were most suitable for LIS implementation in low-resource settings. These 75 statements formed the core functionality requirements for the LIS implementation at KCH.

### System design and development

In our design validation study, we independently assessed BLIS and OpenELIS against our set of 75 functionality requirements for the KCH LIS. The Basic Laboratory Information System met 25 (33%) of the functionality requirements and OpenELIS met 22 (29.3%). A detailed breakdown of the functionality that each system had is presented in Table 1.

**TABLE 1:** Functionality assessment of two open-source laboratory information systems for the Kamuzu Central Hospital laboratory testing process, Malawi, 2015.

LIS-FAT categories	Functionality statements in each testing phase				Systems evaluated							
					Basic Laboratory Information System				Open Enterprise Laboratory Information System			
	PR	AN	PO	CC	PR	AN	PO	CC	PR	AN	PO	CC
Collections and specimen procurement	**3**	**2**	**0**	**1**	1	0	0	1	1	0	0	1
Order entry	**5**	**0**	**0**	**1**	4	0	0	0	4	0	0	0
Test results	**0**	**8**	**7**	**1**	0	2	3	0	0	3	1	0
Verification and auto-verification	**0**	**3**	**0**	**0**	0	2	0	0	0	0	0	0
Worklists	**0**	**2**	**0**	**0**	0	0	0	0	0	0	0	0
Interoperability and data conversion	**0**	**4**	**0**	**2**	0	0	0	1	0	0	0	2
Instruments and handheld devices	**0**	**4**	**0**	**0**	0	0	0	0	0	0	0	0
Labels and barcodes	**1**	**0**	**1**	**1**	0	0	1	1	1	0	0	1
Notifications and warnings	**4**	**5**	**2**	**2**	2	0	0	0	1	1	1	0
Regulations and standards	**0**	**0**	**0**	**1**	0	0	0	1	0	0	0	1
Reports	**0**	**1**	**0**	**2**	0	1	0	2	0	1	0	1
Inventory	**0**	**3**	**0**	**1**	0	0	0	0	0	0	0	0
System downtime	**0**	**1**	**0**	**1**	0	1	0	1	0	0	0	1
Database or technical	**0**	**4**	**0**	**2**	0	1	0	0	0	1	0	0

**Total**	**13**	**37**	**10**	**15**	**7**	**7**	**4**	**7**	**7**	**6**	**2**	**7**

Note: The bold numbers are counts of functionality requirements in each testing phase that the systems were supposed to meet. The non-bold numbers are the actual number of requirements that each system met.

LIS-FAT, Laboratory Information System Functionality Assessment Toolkit; PR, pre-analytical phase; AN, analytical phase; PO, Post-analytical phase; CC, cross-cutting functionality (functionality that affects all the phases and is not restricted to a single phase).

Following the design validation study, BLIS was selected as the base software for the LIS implementation at KCH. To support the pre-analytical and post-analytical phases, we built clinician-facing laboratory order entry and results reporting software modules. Since functionality was already delineated by phases, each phase could be easily conceptualised as an independent component in a larger system. The decision to adopt a modular approach was further driven by the understanding that modules are easier to maintain than a single monolithic system. With the modular approach, any software module can be easily replaced should a more suitable alternative be identified. For example, if another type of LIS software was chosen to replace the customised BLIS at KCH, it could be easily integrated because of the modular approach. This provides flexibility for future improvements of the system. Each module also addressed specific challenges with targeted informatics interventions. A summary of these is provided in Table 2.

**TABLE 2:** Problems in the laboratory testing process and interventions implemented in the laboratory information system to address them at the Kamuzu Central Hospital, Malawi, 2015.

Problem or challenge	Intervention
Use of wrong specimen containers for various tests.	Mapped each test to the correct container type. Picture of correct container is shown to the user when drawing the specimen.
Ordering of multiple tests due to lack of visibility into status of laboratory tests.	List of the patient’s past tests and their status is displayed for review by the clinician.
Failure to test specimens due to inadequate or incomplete documentation.	All the required information was added as mandatory fields for the test ordering process.
Poor specimen viability due to delays in bringing the specimen to the laboratory.	Dashboards added at workstations to provide visual cues on specimens that must be brought to the laboratories and analysed.
Delays in redrawing specimens for orders with missing or non-viable specimens.	Dashboard notification at nursing station when a specimen has been rejected at the laboratory due to non-viability.
Delays in reporting test results.	Dashboard notifications when results are available and the electronic results reporting as soon as the results are entered and verified.
Missing test results.	Electronic results entry allowing multiple and concurrent access to test results.
Failure to analyse specimens due to insufficient volumes of specimen.	Electronic job aid displaying the required volume for each test during specimen drawing.

### Developing bedside solutions

**FIGURE 1:**
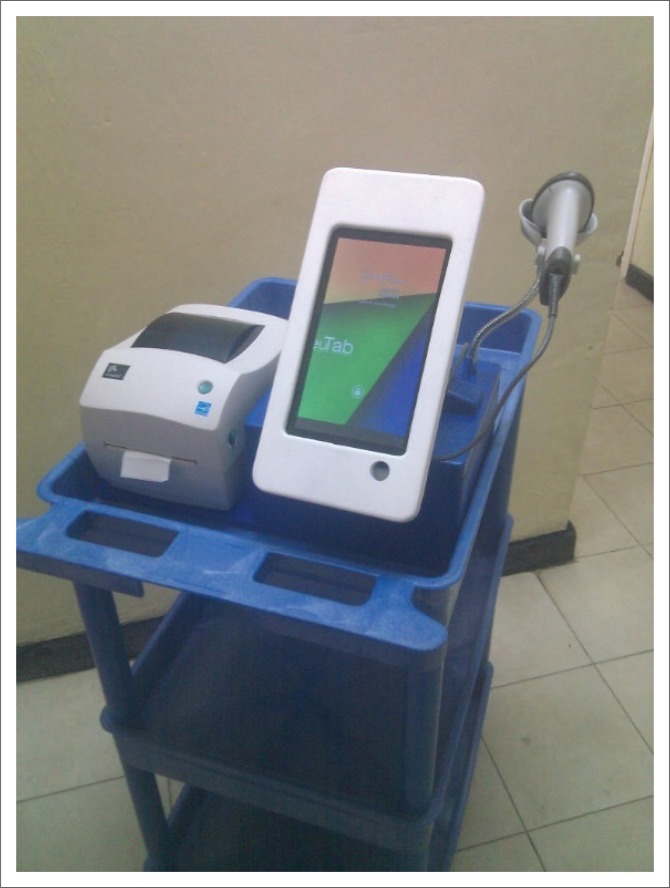
A mobile workstation equipped with a tablet computer, label printer and barcode scanner, Kamuzu Central Hospital, Malawi, 2015.

To facilitate the bedside use of the LIS, we designed custom hardware in the form of a mobile workstation that clinicians could use for ordering laboratory tests and reviewing laboratory results. The mobile workstation is equipped with a low-cost 9-inch tablet computer, a barcode scanner, and a thermal label printer as shown in Figure 1. To provide complete mobility, the workstation is powered by batteries and does not need to be plugged in to a power outlet during use. The batteries are charged between ward rounds when the mobile workstation is not in use. The mobile workstation also provides room for the clinicians and nurses to easily carry around all medical supplies and consumables required for specimen collection during ward rounds. The provision of space for medical supplies was made as a value addition for the medical personnel and eliminated the need for a separate cart for medical supplies.

**FIGURE 2:**
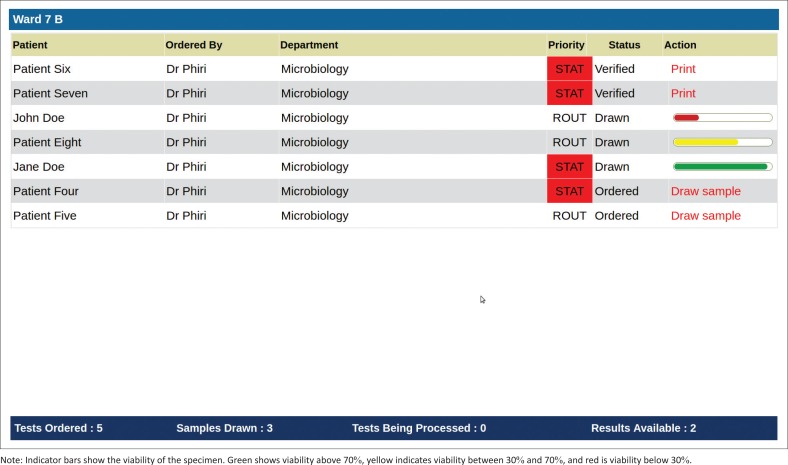
An example of a nursing station dashboard at the Kamuzu Central Hospital, Malawi, 2015.

**FIGURE 3:**
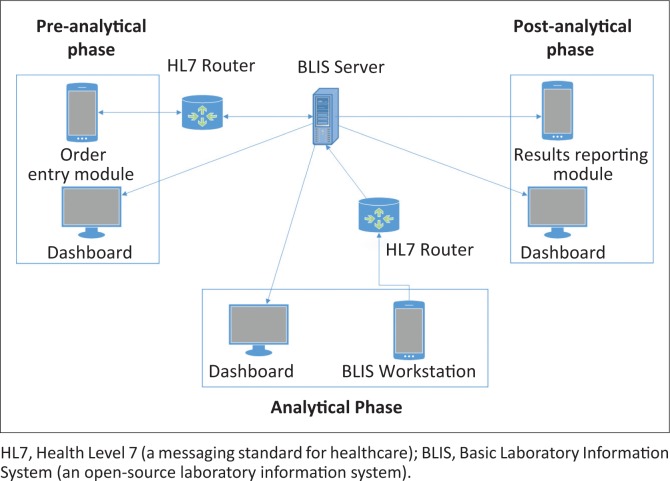
Architecture of laboratory information system implementation at the Kamuzu Central Hospital, Malawi, 2015.

To provide visibility into the status of laboratory tests and results at each stage of the testing process, we also built a dashboard application. This application runs on Raspberry Pi mini-computers connected to 23-inch screens that are mounted in relevant work areas both in the laboratory as well as in the hospital wards. On the screen, we display context-specific work lists such that each user only sees the processes in which they are involved and on which they must act. For example, the dashboard in the microbiology department only shows specimens that require microbiological tests and not any other specimens. A screenshot of the dashboard is provided in Figure 2. Figure 3 depicts how the dashboard application, BLIS and the laboratory order entry and results reporting modules are integrated.

### Laboratory testing workflow with the laboratory information system

The testing process begins with the clinical decision to order a laboratory test to assess the patient’s condition. To initiate an order, the clinician uses a unique national patient identifier in the form of a barcode to open the patient’s record in the Order Entry module. Patient identifiers are issued to patients on arrival at the hospital after completion of a one-time patient registration process, which generates an adhesive label to be affixed to a patient’s personal health passport. A health passport is a paper-based continuity of care document kept by the patient. The framework for uniquely identifying patients at KCH has been described in detail elsewhere.^18^ Scanning the patient’s barcode opens the patient’s summary in the Order Entry module displaying the patient’s past test orders and their status, including test results when available. From this page, the clinician can place new test orders and initiate the pre-analytical phase of the total testing process.

In addition to test ordering, the Order Entry module also maintains and displays an up-to-date catalogue of all the tests that are currently being offered at the facility. This intervention helps prevent ordering of tests that are unavailable in the laboratory. Once the test order has been placed through the Order Entry module, a Health Level 7 message is sent to the BLIS server via a Health Level 7 message router to record the test order. The BLIS server responds by issuing an accession number for the specimen, which is printed on a label in barcode and human readable form together with other test order details during specimen collection. The label is then manually affixed to the specimen container. This accession number is used to track the specimen throughout the testing process. The time at which the specimen label is printed is used in the system as the approximate time when the specimen was collected.

Once the order has been placed, it appears on the relevant nursing station dashboard as well as the laboratory reception dashboard. This was done to provide a visual cue for the laboratory receptionist to anticipate incoming specimens from the ward while at the same time reminding nursing station staff that they need to collect and transport the specimens to the laboratory. Once a specimen has been collected, the dashboard displays its viability based on how long it has been since it was drawn. This serves as a reminder for the nursing staff to bring the specimens to the laboratory on time. The specimen will continue to show on the nursing station dashboard until it has been received at the laboratory reception.

When the specimen arrives at the laboratory, the laboratory receptionist scans the barcode and performs a visual inspection of the specimen container and test order documentation. Based on this, the receptionist determines whether the specimen should be accepted or rejected. For example, a specimen can be rejected if it is no longer viable depending on when it was drawn and the type of test that was ordered. When a specimen is rejected, a notification appears on the nursing station dashboard informing the nursing staff to redraw the specimen. If the specimen is accepted at laboratory reception, it is sent to the appropriate laboratory department for analysis and an entry is added to that department’s dashboard. This is the beginning of the analytical phase. The department dashboard acts as a dynamic worklist informing laboratory technicians of tests that need to be run and results that have yet to be recorded.

Once a specimen has been analysed, the laboratory technician enters the result using a touchscreen workstation in the laboratory department to complete the analytical phase of the testing process. The nursing staff are notified of the new result through the nursing station dashboard and can now print out the test result and affix it to the patient’s health passport or medical chart for review by the clinician.

## Discussion

In this article, we have described the process used to implement a LIS in a low-resource setting, specifically at KCH in Lilongwe, Malawi. We aimed to demonstrate a problem-driven approach that implements individual informatics interventions to support the laboratory testing process in a low-resource setting. We demonstrated this by piloting a system that supported the entire testing process for outpatient tuberculosis screening tests at KCH.

An example of a problem that was addressed in this implementation is the incorrect use of specimen containers for various tests. To address this problem, a picture of the correct container and required specimen volume for each test type was presented to the users during specimen drawing as an electronic job aid. These two interventions addressed the cause of 84% of all untestable specimens at KCH that we reported in a previous article.^13^

While the pilot implementation in the outpatient tuberculosis clinic achieved our main goal, it also limited our ability to measure the impact of the interventions. For instance, sputum is the only specimen type collected in the outpatient tuberculosis clinic at KCH. Therefore, we could not measure the impact of the specimen container decision support on specimen viability. Furthermore, since this ward serves outpatients, the laboratory turnaround time for these tests is not an accurate measure of process efficiency as the review of the results depends on when the patient returns to the hospital, and not when the actual test result itself becomes available. Despite these limitations, there are several points worth noting from this work.

A set of 75 functionality requirements (Online Appendix 1) for LIS in low-resource settings was produced as part of this project. This contains significantly fewer requirements than the 850 statements found in the LIS-FAT document, which are more appropriate for high-resource settings. These 75 requirements can be used by other implementers in low-resource settings to design and evaluate their own LIS.

A further point can be made about the actual design of the implementation. The use of distinct modules separated by the phases of the total testing process offers several benefits. Not only can the modules be more easily maintained, they can also be easily replaced should better alternatives be identified. This offers significant benefits going forward as the implementation is not tightly coupled to any single piece of software.

This implementation further highlights the benefits that open-source software provides with regard to systems implementation in low-resource settings. Software development takes time and is expensive. However, using existing open-source software has the potential to vastly reduce both effort and cost. For instance, design and development took only 10 weeks because existing software was reused for some parts of our system. This could have been significantly longer if everything was built from scratch. Therefore, we recommend that other implementers in low-resource settings find ways of making use of the many open-source products in the health informatics community as this can reduce their effort and expenditure.

Using cheaper alternatives is a common approach to cost reduction. In this implementation, we did this by using tablets that cost $60.00 for the workstations instead of the $650.00 touchscreen clinical workstations that we have used in the past. The tablets presented a significant price reduction and other desirable qualities like the ability to easily be mounted on the mobile workstation. There seemed to be no significant problems with the tablets during the testing phase. However, when we deployed them in the hospital, the tablets often stopped responding and would occasionally power down during use without warning. This led to the loss of all current work that clinical staff had done related to the current patient or specimen and was very disruptive to the workflow.

Our experience with the tablets emphasises the need for rigour in the testing of new hardware before deployment. In the next iteration, we will address this by replacing the tablets with Raspberry Pi mini-computers and 10.1-inch touchscreen displays. We have comprehensively tested this solution and believe that these new computers will perform more reliably than the tablets and will not significantly inflate the expenditure on the project as they cost less than $200.00 each.^19^

The main limitation of this implementation was our inability to measure the impact that the interventions had on various laboratory key performance indicators such as turnaround times for laboratory tests. In addition, we did not deploy the mobile workstation in the inpatient wards for use during ward rounds. This was mainly due to dependencies that had to be met before deploying the system to inpatient wards at KCH. In the future, we intend to perform field usability evaluations for the mobile workstations and problem impact studies to quantify the effect of the various interventions on laboratory key performance indicators.

Lessons learnt from this pilot have informed the continuing scale-up of LIS implementations in Malawi. A revised version of this system has now been deployed in three central hospitals and four district hospitals.^20^ Revisions have focused on improving operations in the analytical phase by interfacing instruments to the LIS. Future efforts will focus on maximising the benefits in the pre-analytical and post-analytical phases where most laboratory errors occur.

Lessons learned

- An electronic laboratory information system that supports the entire testing process can work in the absence of an electronic medical records system.
- Leveraging individual interventions to solve systemic process challenges can provide benefits to clinical staff that incentivise them to continue using the interventions after implementation.
- Pre-existing functionality assessment toolkits can provide a foundation for building better tools to improve workflows and processes.
